# HIV and women’s health: Where are we now?

**DOI:** 10.1177/17455065221076341

**Published:** 2022-02-02

**Authors:** Shema Tariq, Bakita Kasadha

**Affiliations:** 1 UCL Centre for Clinical Research in Infection and Sexual Health, Institute for Global Health, University College London, London, UK; 2 Mortimer Market Centre, CNWL NHS Foundation Trust, London, UK; 3 Nuffield Department of Primary Care Health Sciences, University of Oxford, Oxford, UK

*‘Women get AIDS too.*

*Women are being ignored in the AIDS crisis.*

*Women need healthcare services.*

*Women need childcare.*

*Women need access to AIDS treatments.*

*Women need access to drug rehab treatment programs.*

*Women need safer sex information and men need to use condoms.*

*The city is not giving women what they need.*

*Demand to know why!’*

ACT UP New York Poster, circa late 1980s/early 1990s

Women have always been here in the HIV pandemic, as patients, as researchers, as doctors, as nurses, as mothers, as carers, as lovers and as activists. It is often forgotten that, among the 70 cases of what we now know to be HIV reported by the Centers for Disease Control in August 1981, was one woman.^1^ By late 1982, reports of infections in infants emerged, suggesting for the first time that HIV could be transmitted vertically.^2^ A few months later, we learned about cases among female sexual partners of men with HIV.^3^

As we enter the fifth decade of the pandemic, 53% of the 37.7 million people living with HIV globally are women and girls.^4^ In sub-Saharan Africa, six out of seven new HIV infections among 15- to 19-year-olds are among girls.^4^ Around 4200 women aged 15–24 years in sub-Saharan Africa acquired HIV every week in 2020.^4^ The risk of acquiring HIV is 34 times higher in trans women than cisgender women; sex workers are 26 times greater risk.^4^ Sex workers who use drugs are particularly vulnerable to acquiring HIV, with high prevalence reported in parts of Eastern Europe and Central Asia.^5–8^ Despite dramatic declines in rates of vertical transmission, mainly due to combination prevention approaches that include antenatal HIV testing and antiretroviral therapy (ART) for pregnant and breastfeeding women, there were 150,000 new infections in children aged 14 and under in 2020,^4^ with evidence that progress is slowing especially in some regions of sub-Saharan Africa. HIV pre-exposure prophylaxis (PrEP), although highly effective, remains under-utilized among women due to limited guidelines, lack of targeted education and challenges with adherence.^9^ In addition, criminalization of HIV transmission via sex and breastfeeding and state intervention undermine access to HIV prevention, HIV care and retention in care, contexts in which support is more likely to keep women and their families well.^10,11^

In the United Kingdom, where we both live, nearly one-third of the 98,000 people seen for HIV care in 2019 were women.^12^ That same year, just over a 1000 women were newly diagnosed with HIV, continuing the downwards trend in new diagnoses.^13^ However, the decline in new HIV infections has not been as steep as that in gay and bisexual men, highlighting an important disparity in the provision and uptake of combination prevention.^13^ Despite high uptake of HIV testing antenatally, women are less likely to have an HIV test in sexual health services (the main setting for HIV testing other than antenatal screening) than gay and bisexual men; over half of women diagnosed with HIV in 2017 in the United Kingdom were diagnosed late (CD4 cell count <350 cells/mm^3^),^14^ a pattern observed across Europe.^15^ Furthermore, across parts of sub-Saharan Africa, studies have revealed significant barriers to HIV testing among married women; among adolescents, removing age-requirements for consenting to HIV testing has been shown to improve testing rates.^16–18^

Yet women remain largely under-represented in HIV research. A 2016 systematic review found that only 20% of antiretroviral trial participants were women; an even smaller proportion (10%) were recruited into HIV cure trials.^19^ In the landmark 2018 ‘Invisible No Longer’ report on HIV and women in the United Kingdom, women living with HIV highlight this lack of visibility and involvement in research and policy-making as a key concern.^20^ This under-representation is most marked among key populations including women who inject drugs, a growing population who encounter gender-related barriers to harm reduction services and research participation.^5,6,8^ Of course, this gender inequity extends beyond HIV into clinical research more broadly, largely resulting from a historical neglect of sex and gender differences in pathophysiology and drug activity, as well as concerns around the inclusion of women of childbearing potential.^21,22^ We have seen this play out most recently during the COVID-19 pandemic, where the lack of trial data has led to vaccine hesitancy among pregnant women despite the increased risk of severe disease, pregnancy complications and stillbirth.^23^ Women living with HIV (many of whom are from socially marginalized groups) may face additional barriers to research participation such as language barriers, HIV-related stigma, medical mistrust and practical considerations such as work, caring responsibilities and transport costs.^24^

Without data specifically focusing on women living with HIV, we are limited in our ability to provide appropriate care and support. Experiences of HIV are inherently gendered, both biologically and socially. For instance, although antiretrovirals have been shown to be equally effective in men and women, women have been reported to have an increased risk of side effects with some antiretrovirals compared to men, such as rashes with etravirine and weight gain with dolutegravir.^25,26^ This may partly explain why women are more likely than men to report treatment discontinuation.^27^ Furthermore, there are important considerations such as contraception, pregnancy, menopause and gender re-affirming hormone therapy that both shape women’s experiences of living with HIV, and their management including antiretroviral choice. In the case of pregnancy, the exclusion of pregnant women from the dolutegravir-licensing trials led to prolonged uncertainty about potential risks of neural tube defects, with many women encouraged to switch from this highly efficacious medication when the risk is likely to have been negligible.^28^

Of even greater concern is the failure to uphold the sexual and reproductive health rights of women living with HIV within healthcare settings, ranging from coercion to share HIV status with partners, to forced sterilization and obstetric violence.^29^ Human rights must be ensured while protecting health. Women living with HIV often also encounter multiple and intersecting social disadvantage including gender-based violence (disproportionately impacting trans women), poverty, poor mental health and immigration issues,^14,30,31^ all of which increase their vulnerability within healthcare settings as well as impacting their long-term health and well-being. HIV research must engage with the plurality of these experiences.

And what about celebrating strengths and achievements? Rates of vertical transmission of HIV have declined dramatically in many parts of the world. Women with HIV are now living longer, and, for the first time in the history of the HIV pandemic, are reaching middle and later life in great numbers. Women living with HIV are at the forefront of community mobilization for services, delivery of peer support, and advocacy for their rights and women-centred HIV prevention (such as the dapivirine ring), care and research. Women living with HIV have demanded to be seen beyond their childbearing capacity, and to have access to all available treatment options. They continue to advocate for increased choice regarding prevention and treatment options, and to be heard. This is all testament to their strength and resilience.

We are, therefore, delighted to introduce this special collection across Women’s Health and Therapeutic Advances in Infectious Disease dedicated to women and HIV. Our aim is to provide an overview of leading-edge work engaging with cis- and transgender women and HIV, from prevention, to management, to lived experiences. Importantly, we set out to be inclusive of all methodologies, driven by our belief that there are multiple ways of producing knowledge. This has resulted in a collection that spans the breadth of approaches from arts and humanities, to social sciences, to epidemiological research. We specifically wanted to broaden our scope beyond the traditional focus on HIV and pregnancy, in recognition that women’s lives are not defined exclusively by reproduction. Reflecting our commitment to the meaningful inclusion of women living with HIV in the production of knowledge, this special collection is co-edited by a woman living with HIV, a rare occurrence in academic publishing; we hope this sets a precedent. We have actively sought submissions from women with lived experience; consequently, several papers in this collection are authored by women living with HIV. However, this special collection is an open access publication that does not permit anonymous submissions; we therefore acknowledge that women who are not living openly with HIV may have been reluctant to contribute. In developing this collection, we have strived to embody the ethos of women living with HIV, to honour all people living with HIV by promoting the use of stigma free language,^32^ and to reflect the multiple and diverse experiences from across the globe.

This special collection spans the globe from high-income settings such as the United Kingdom and the United States, to lower-income settings such as Nepal and Mali. It explores the breadth of the HIV prevention and care continuum, and highlights how factors such as race and ethnicity, age, gender and HIV status intersect to shape women’s lives. We invite you to engage with this multiplicity of experiences. Forty-one years into the HIV pandemic, women are to be ignored no longer.
